# Transferability of interatomic potentials for silicene

**DOI:** 10.3762/bjnano.14.48

**Published:** 2023-05-08

**Authors:** Marcin Maździarz

**Affiliations:** 1 Department of Computational Science, Institute of Fundamental Technological Research Polish Academy of Sciences, Pawińskiego 5B, 02-106 Warsaw, Poland

**Keywords:** 2D materials, DFT, force fields, interatomic potentials, mechanical properties, silicene

## Abstract

The ability of various interatomic potentials to reproduce the properties of silicene, that is, 2D single-layer silicon, polymorphs was examined. Structural and mechanical properties of flat, low-buckled, trigonal dumbbell, honeycomb dumbbell, and large honeycomb dumbbell silicene phases, were obtained using density functional theory and molecular statics calculations with Tersoff, MEAM, Stillinger–Weber, EDIP, ReaxFF, COMB, and machine-learning-based interatomic potentials. A quantitative systematic comparison and a discussion of the results obtained are reported.

## Introduction

We are living in the “silicon age” because of the great importance of elemental silicon to the modern global economy, mainly concerning electronics. Silicon is one of the most extensively investigated materials, and the quality of its production is impressive. However, this applies to bulk silicon. The success of graphene has also sparked interest in other non-carbon 2D materials [1–2]. One of such materials is 2D silicon, called silicene [3–4]. Using first-principles methods with current computer resources enables us to model structures up to about a few hundred atoms. For larger systems, approximate methods are needed, for example, molecular dynamics/statics. For these methods, the quality of the used interatomic potentials (IAPs) is crucial. Because of the importance of silicon, as well as its complexity, dozens of potentials have been proposed for it. In the very well-known NIST Interatomic Potentials Repository, there are 27 potentials for silicon (the highest number of potentials provided for a chemical element), the oldest one from 1985 and the latest from 2020 [5–6].

At least five 2D silicon polymorphs have been reported in the literature, that is, flat (FS), low-buckled (LBS) [7], trigonal dumbbell (TDS), honeycomb dumbbell (HDS), and large honeycomb dumbbell (LHDS) silicene [8]. There are still doubts about their dynamic stability. For example, for a flat phase, the negative ZO phonon mode could be removed by the selection of an appropriate substrate [3,9]. The ability of potentials for 3D silicon to reproduce 2D silicon is poorly studied. There are several papers in which the quality of potentials for 3D silicon has been assessed [10–12], but not for silicene. The intention of this work is first to determine the structural and mechanical properties of 2D silicon using the first-principles method and then to test the ability of different interatomic potentials to reproduce these properties.

## Methods

Analyzing the available literature concerning all phases of single-layer Si, it is not feasible to find all structural, mechanical, and phonon data obtained in one consistent way. The availability of experimental data is actually limited to the silicene grown on supports, a pristine free-standing single-layer sheet of silicene has not yet been discovered [4,13]. Therefore, we must use ab initio calculations. Unfortunately, also ab initio calculations, most often DFT, differ in the calculation methodology, that is, they use different functional bases and different pseudopotentials or exchange–correlation functionals. Also, parameters such as cohesive energy and elastic constants are poorly accessible. For this reason, structural and mechanical data, that is, lattice parameters, average cohesive energy, average bond length, average height, 2D elastic constants, as well as phonon data are determined here using a single consistent first-principles approach as described in the next section “Ab initio calculations”. These data were further considered as reference data and marked as “value^DFT^”. Then, the same data were determined as described in section “Molecular calculations” using the analyzed molecular potentials from subsection “Interatomic potentials” and are marked as “value^potential^”. Having both data, we can simply define the mean absolute percentage error (MAPE):


[1] $$\text{MAPE}=\frac{100\%}{n}\sum_{t=1}^{n}\left|\frac{{\text{value}}^{\text{DFT}}-{\text{value}}^{\text{potential}}}{{\text{value}}^{\text{DFT}}}\right|,$$

which enables us to quantify the potentials under examination.

For 2D materials, directional 2D Young’s moduli,


[2] $$E_{\left[10\right]}^{2\text{D}}=\frac{C_{11}C_{22}-C_{12}^{2}}{C_{22}}\text{ and }E_{\left[01\right]}^{2\text{D}}=\frac{C_{11}C_{22}-C_{12}^{2}}{C_{11}},$$

2D Poisson’s ratios,


[3] $$\nu_{\left[10\right]}^{2\text{D}}=\frac{C_{12}}{C_{22}}\text{ and }\nu_{\left[01\right]}^{2\text{D}}=\frac{C_{12}}{C_{11}},$$

and the 2D shear modulus,


[4] $$G^{2\text{D}}=C_{33},$$

are often used instead of elastic constants *C*_ij_. Because of the symmetry of hexagonal lattices, these reduce to one 2D Young’s modulus *E* and one 2D Poisson’s ratio ν [14].

### Ab initio calculations

The ab initio calculation methodology here is closely analogous to that used in [15]. Hence, its description is also very similar, that is, density functional theory (DFT) [16–17], ABINIT plane-wave approximation code [18–19], local density approximation (LDA) [20–21] as an exchange–correlation functional, and optimized norm-conserving Vanderbilt pseudopotential [22] (ONCVPP) are similar. Cut-off energy and electron configuration of Si were used in the DFT calculations according to the pseudopotential and Gaussian smearing scheme with *tsmear* (Ha) = 0.02. To generate k-points grids, *kptrlen* was set to 43.0. Since the 2D structures were analyzed in the *z* direction, a vacuum of 20 Å was applied. The initial data for the five structures analyzed were deduced from [7] and [8]. The structures were then carefully relaxed with full optimization of cell geometry and atomic coordinates [15].

The average cohesive energy *E*_c_ (eV/atom) was computed as the difference in the total energy of a given relaxed earlier structure and that of its individual atoms placed in a cubic box of sufficient size. The theoretical ground state, *T* = 0 K, and the elastic constants, *C*_ij_, of all previously optimized structures were computed using the metric tensor formulation of strain in the density functional perturbation theory (DFPT) [23]. The mechanical stability of the analyzed structures was verified by calculating the so-called Kelvin moduli [24–25]. To calculate the phonons, the DFPT implemented in ABINIT [18–19] was employed. The phonon dispersion curves along the path **Γ**[0,0,0]–**M**[1/2,0,0]–**K**[1/3,1/3,0]–**Γ**[0,0,0] [26] of the analyzed structures were then used to identify their dynamical stability [27], complementary to the mechanical stability.

### Molecular calculations

To perform molecular calculations, the molecular statics (MS) method, *T* = 0 K [28–30], was used by means of the “large-scale atomic/molecular massively parallel simulator” (LAMMPS) [31] and analyzed by means of the “Open Visualization Tool” (OVITO) [32].

As for DFT calculations, the structures here were fully pre-relaxed with the conjugate gradient (CG) algorithm and, then, the elastic constants, *C*_ij_, were calculated for them using the stress–strain method with the maximum strain magnitude set to 10^−6^ [30–31]. In the *z* direction, a vacuum was set to 20 Å.

To measure the performance of the analyzed interatomic potentials, a series of molecular dynamics (MD) simulations (200 atoms and 10000 timesteps, NVE ensemble) and LAMMPS’s built-in function timesteps/s were used. The results were then normalized relative to the longest run time.

#### Interatomic potentials

The parameterizations of the potentials listed below were obtained from the NIST Interatomic Potentials Repository and/or from LAMMPS code sources.

1. Tersoff1988 [33]: the original Tersoff potential for silicon (it is important to remember that this paper proposed a form of potential rather than a specific parametrization for silicon)
2. Tersoff2007 [34]: the Tersoff potential fitted to the elastic constants of diamond silicon
3. Tersoff2017 [35]: newer, better optimized the Tersoff potential for silicon
4. MEAM2007 [36]: the semi-empirical interatomic potential for silicon based on the modified embedded atom method (MEAM) formalism
5. MEAM2011 [37]: the spline-based modified embedded-atom method (MEAM) potential for Si fitted to silicon interstitials
6. SW1985 [38]: the Stillinger–Weber (SW) potential fitted to solid and liquid forms of Si
7. SW2014 [39]: the Stillinger–Weber (SW) potential fitted to phonon dispersion curves of a single-layer Si sheet
8. EDIP [40]: the environment-dependent interatomic potential (EDIP) fitted to various bulk phases and defect structures of Si
9. ReaxFF [41]: the reactive force-field (ReaxFF) fitted to a training set of DFT data that pertain to Si/Ge/H bonding environments
10. COMB [42]: the charge optimized many-body (COMB) potential fitted to a pure silicon and five polymorphs of silicon dioxide
11. SNAP [43]: the machine-learning-based (ML-IAP) linear variant of spectral neighbor analysis potential (SNAP) fitted to total energies and interatomic forces in ground-state Si, strained structures, and slab structures obtained from DFT calculations
12. qSNAP [43]: the machine-learning-based (ML-IAP) quadratic variant of spectral neighbor analysis potential (qSNAP) fitted to total energies and interatomic forces in ground-state Si, strained structures, and slab structures obtained from DFT calculations
13. SO(3) [44]: the machine-learning-based (ML-IAP) variant of the SO(3) smooth power spectrum potential (SO(3)) fitted to the ground-state of the crystalline silicon structure, strained structures, slabs, vacancy, and liquid configurations from DFT simulations
14. ACE [45]: the machine-learning-based (ML-IAP) variant of the atomic cluster expansion potential (ACE) fitted to a wide range of properties of 3D silicon determined from DFT calculations

## Results and Discussion

### Structural and mechanical properties

**Figure 1:**
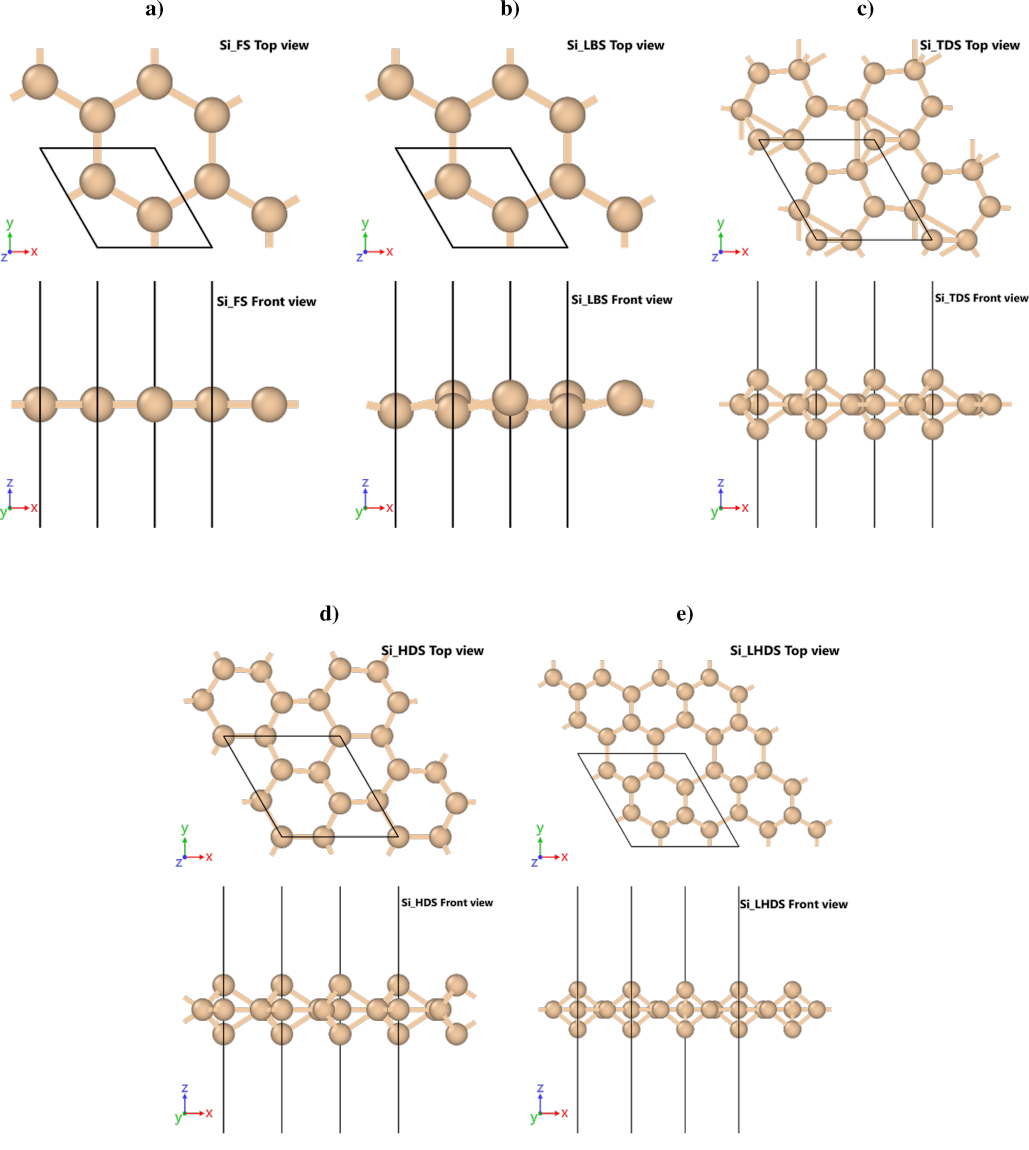
Polymorphs of silicene: (a) flat (FS): *hP*2, *P*6/*mmm*, no.191, (b) low-buckled (LBS): *hP*2, 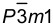, no.164, (c) trigonal dumbbell (TDS): *hP*7, 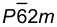, no.189, (d) honeycomb dumbbell (HDS): *hP*8, *P*6/*mmm*, no.191, (e) large honeycomb dumbbell (LHDS): *hP*10, *P*6/*mmm*, no.191.

Basic cells for the five silicene polymorphs, that is, flat (FS) (*hP*2, P6/mmm, no.191), low-buckled (LBS) (*hP2*, 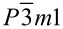, no.164), trigonal dumbbell (TDS) (*hP*7, 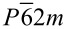, no.189), honeycomb dumbbell (HDS) (*hP*8, *P*6/*mmm*, no.191), and large honeycomb dumbbell (LHDS) (*hP*10, P6/mmm, no.191), are depicted in Figure 1. Additionally, the crystallographic data for them are stored in crystallographic information files (CIFs) in Supporting Information Files 1–5.

The results of first-principles calculations show that all silicene phases have hexagonal symmetry. The symmetry characteristics of a structure determine the symmetry of its physical properties (cf. Neumann’s Principle and Curie laws) [30,46]. For 2D linear hyperelastic materials, there are four classes of symmetry [25], and hexagonal symmetry implies isotropy of the stiffness tensor, that is, there are only two distinct elastic constants and they satisfy, in Voigt notation, such conditions that *C*_11_ = *C*_22_, *C*_33_ = (*C*_11_ − *C*_12_)/2.

Structural and mechanical characteristics that were determined from DFT computations, namely lattice parameters, average cohesive energy, average bond length, average height, 2D elastic constants, 2D Young’s modulus, Poisson’s ratio, and 2D Kelvin moduli, of the five silicene polymorphs analyzed are gathered in Table 1. Since we are analyzing free-standing silicene here, which has not yet been observed in experiments, we compare the results of the calculations with those of other authors. We find that the lattice constants, average bond length, average height and cohesive energy agree at the DFT level of accuracy with other calculations. Mechanical properties of silicene are available in the literature only for the LBS phase and are limited to 2D Young’s modulus and Poisson’s ratio only. These quantities are also in reasonable agreement with the present results. It is worth noting that all calculated 2D Kelvin moduli for all silicene phases are positive, which results in mechanical stability [25].

**Table 1:** Structural and mechanical properties of flat (FS), low-buckled (LBS), trigonal dumbbell (TDS), honeycomb dumbbell (HDS), and large honeycomb dumbbell (LHDS) silicene phases from density functional theory (DFT) calculations: lattice parameters *a* and *b* (Å), average cohesive energy *E*_c_ (eV/atom), average bond length *d* (Å), average height *h* (Å), 2D elastic constants *C*_ij_ (N/m), 2D Young’s modulus *E* (N/m), Poisson’s ratio ν, and 2D Kelvin moduli *K*_i_ (N/m).

Polymorph	FS		LBS		TDS		HDS		LHDS	

Source	This work	Refs.	This work	Refs.	This work	Refs.	This work	Refs.	This work	Refs.

*a*	3.855	3.90^a^	3.828	3.87^a^, 3.83^b^	6.434	6.52^c^	6.297	6.38^c^	7.334	7.425^c^
*b*	3.855	3.90^a^	3.828	3.87^a^, 3.83^b^	6.434	6.52^c^	6.297	6.38^c^	7.334	7.425^c^
−*E*_c_	4.562	4.764^a^	4.577	4.784^a^, 5.16^b^	4.679		4.679		4.769	
*d* ^d^	2.225		2.249	2.25^b^	2.331		2.399		2.357	
*h*	0.0	0.0^a^	0.421^a^	0.45^a^, 0.44^b^	2.734		2.635		2.683	
*C*_11_	84.8		69.2		100.5		141.6		104.5	
*C*_22_	84.8		69.2		100.5		141.6		104.5	
*C*_12_	40.6		22.1		52.3		96.4		52.7	
*C*_33_	22.1		23.6		24.1		22.6		25.9	
*E*	65.4		62.2	61.8^a^	73.3		76.0		77.9	
ν	0.48		0.32	0.31^a^	0.52		0.68		0.50	
*K*_I_	125.4		91.3		152.8		238.0		157.2	
*K*_II_	44.3		47.1		48.3		45.2		51.8	
*K*_III_	44.3		47.1		48.3		45.2		51.8	

^a^Ref. [47], ^b^Ref. [7], ^c^Ref. [8], ^d^average bond lengths calculated using a radial pair distribution function with a cut-off radius of 3.0 Å and a number of histogram bins of 1000 [32].

**Figure 2:**
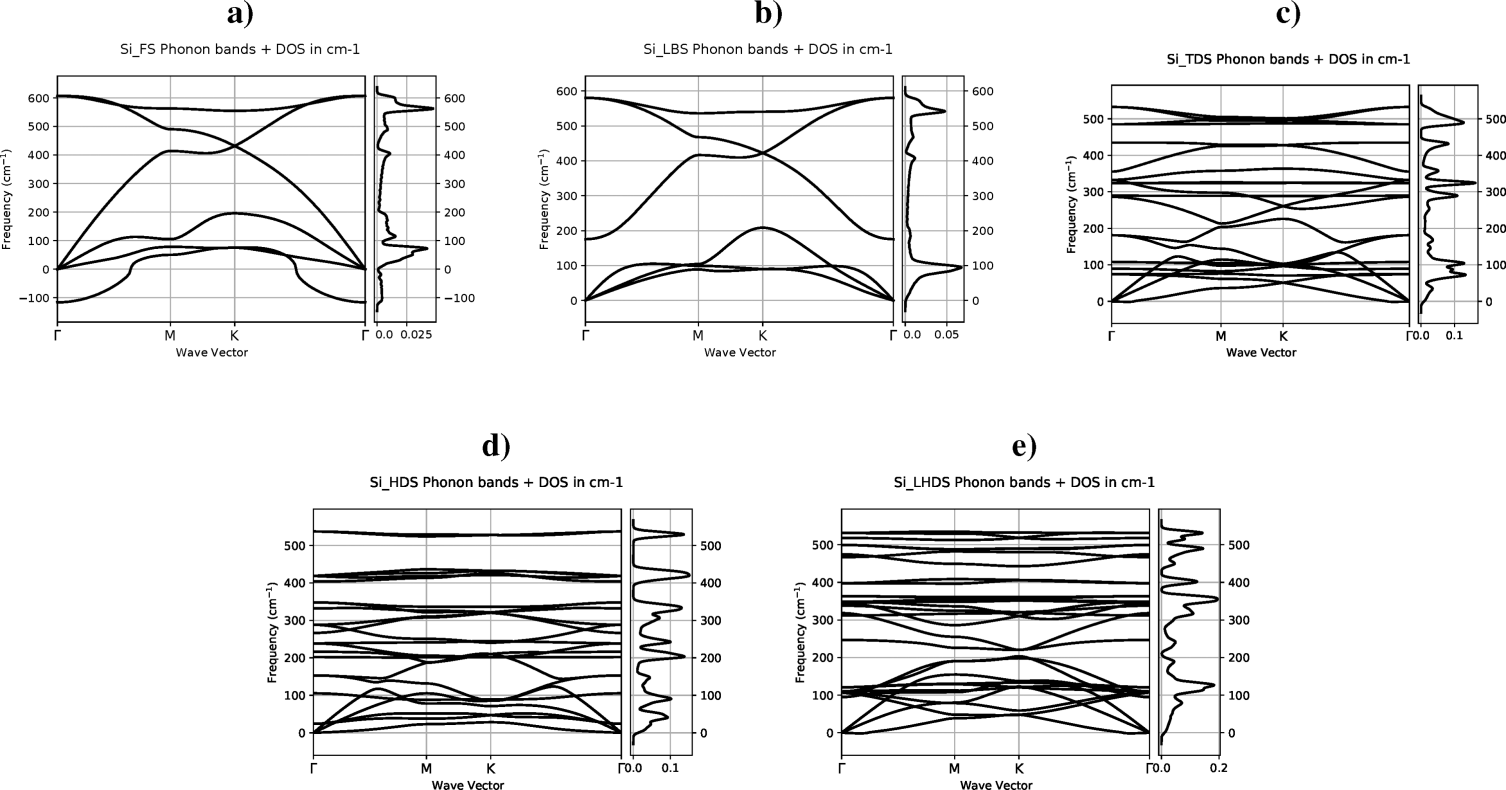
Phonon dispersion and density of states (DOS): (a) the flat (FS), (b) low-buckled (LBS), (c) trigonal dumbbell (TDS), (d) honeycomb dumbbell (HDS), and (e) large honeycomb dumbbell (LHDS) single-layer silicon (silicene) phases. High symmetry points: **Γ**[0,0,0], **M**[1/2,0,0], and **K**[1/3,1/3,0].

The phonon spectra along the **Γ**-**M**-**K**-**Γ** path for the five silicene polymorphs is depicted in Figure 2. The analysis of the computed curves shows that the phases TDS, LBS, HDS, and LHDS are not only mechanically but also dynamically stable, that is, all phonon modes have positive frequencies anywhere. The FS phase is mechanically stable, but can be dynamically unstable, that is, the optical ZO phonon mode has a negative frequency. Other authors have also observed similar FS phase behavior [4,13]. However, since silicene is not a free-standing structure in nature, the selection of a proper substrate may dampen the out-of-plane vibration mode, and flat silicene may be produced [3]. Hence, it was decided to include also this phase in the molecular calculations.

### Performance of interatomic potentials

Computations were carried out using molecular statics and the fourteen interatomic potentials for silicon, (Tersoff (×3), MEAM (×2), Stillinger–Weber (×2), EDIP, ReaxFF, COMB and machine-learning-based (ML-IAP (×4)), enumerated in section “Interatomic potentials”. Twelve structural and mechanical properties, that is, lattice parameters *a* and *b*, average cohesive energy *E*_c_, average bond length *d*, average height *h*, 2D elastic constants *C*_ij_, and 2D Kelvin moduli *K*_i_, are collected in Table 2 for flat silicene (FS), in Table 3 for low-buckled silicene (LBS), in Table 4 for trigonal dumbbell silicene (TDS), in Table 5 for honeycomb dumbbell silicene (HDS), and in Table 6 for large honeycomb dumbbell silicene (LHDS), respectively. The aforementioned results, for each of the five silicene phases, were then compared with those from DFT calculations using the mean absolute percentage error (MAPE) defined in Equation 1. Let us briefly analyze the results for each phase. For the FS phase, the most accurate is the MEAM2011 potential as it has the lowest MAPE , see Table 2. For the LBS phase, it is the Tersoff2107 potential, see Table 3. For the TDS phase, it is the ReaxFF potential, see Table 4. For the HDS phase, it is the ReaxFF potential, see Table 5, and finally, for the LHDS phase, it is again the ReaxFF potential, see Table 6. Now let us take a summary look. Seven of the analyzed fourteen potentials, namely Tersoff2007, Tersoff2017, SW1985, SW2014, ReaxFF, SNAP, and ACE, are able to correctly reproduce the structural properties of the five polymorphs of silicene, see Table 3, Table 5, and Table 6. Two potentials, ReaxFF and MEAM2011, give the best quantitative performance measured by the total mean absolute percentage error (MAPE), see Table 6. Regarding the cost of calculations in terms of relative performance measured as normalized timesteps per second in molecular dynamics (MD) simulations, the EDIP and Stillinger–Weber potentials are the fastest, about five times faster than the MEAM and Tersoff potentials, about 100 times faster than ReaxFF and COMB, and up to 2000 times faster than the ML-IAP(ACE) potential, see Table 6. It is also worth noting, that the machine-learning-based (ML-IAP) interatomic potentials, according to the methodology used, are not superior to classical potentials in terms of performance (MAPE) and are instead even three orders of magnitude more computationally expensive, see Table 6.

**Table 2:** Structural and mechanical properties of flat silicene (FS) from molecular calculations: lattice parameters *a* and *b* (Å), average cohesive energy *E*_c_ (eV/atom), average bond length *d* (Å), average height *h* (Å), 2D elastic constants *C*_ij_ (N/m), 2D Kelvin moduli *K*_i_ (N/m), and mean absolute percentage error (MAPE) (%).

Method	DFT	Tersoff1988	Tersoff2007	Tersoff2017	MEAM2007	MEAM2011	SW1985	SW2014

*a*	3.855	4.008	4.019	4.042	4.457	3.960	4.104	3.886
*b*	3.855	4.008	4.019	4.042	4.457	3.960	4.104	3.886
−*E*_c_	4.562	3.926	3.828	3.687	3.288	3.793	3.145	2.564
*d*	2.225	2.315	2.321	2.333	2.573	2.288	2.369	2.243
*h*	0.0	0.0	0.0	0.0	0.0	0.0	0.0	0.0
*C*_11_	84.8	54.6	55.2	47.7	57.3	84.0	58.8	57.3
*C*_22_	84.8	54.6	55.2	47.7	57.3	84.0	58.8	57.3
*C*_12_	40.6	49.5	47.4	52.8	29.7	40.8	34.4	33.0
*C*_33_	22.1	2.6	3.9	−2.6	13.8	21.6	12.2	12.2
*K*_I_	125.4	104.0	102.6	100.5	87.0	124.8	93.2	90.3
*K*_II_	44.3	5.1	7.8	−5.1	27.6	43.3	24.4	24.4
*K*_III_	44.3	5.1	7.8	−5.1	27.6	43.3	24.4	24.4
MAPE_FS_		36.515	34.619	45.987	28.174	3.147	26.093	26.595

Method		EDIP	ReaxFF	COMB	ML-IAP SNAP	ML-IAP qSNAP	ML-IAP SO(3)	ML-IAP ACE

*a*		4.018	3.950	3.990	4.121	4.019	4.051	3.850
*b*		4.018	3.950	3.990	4.121	4.019	4.051	3.850
−*E*_c_		4.010	3.408	3.911	4.575	4.499	4.407	0.962
*d*		2.321	2.282	2.306	2.381	2.321	2.340	2.222
*h*		0.0	0.0	0.0	0.0	0.0	0.0	0.0
*C*_11_		87.4	74.7	79.7	41.5	24.7	41.0	88.9
*C*_22_		87.4	74.7	79.7	41.5	24.7	41.0	88.9
*C*_12_		9.2	36.8	23.9	16.2	17.6	23.9	44.8
*C*_33_		39.1	19.0	27.9	12.6	3.5	8.6	22.0
*K*_I_		96.6	111.5	103.6	57.6	42.3	64.9	133.7
*K*_II_		78.2	37.9	55.8	25.3	7.0	17.1	44.1
*K*_III_		78.2	37.9	55.8	25.3	7.0	17.1	44.1
MAPE_FS_		32.827	10.904	15.805	33.284	48.283	35.946	9.757

**Table 3:** Structural and mechanical properties of low-buckled silicene (LBS) from molecular calculations: lattice parameters *a* and *b* (Å), average cohesive energy *E*_c_ (eV/atom), average bond length *d* (Å), average height *h* (Å), 2D elastic constants *C*_ij_ (N/m), 2D Kelvin moduli *K*_i_ (N/m), and mean absolute percentage error (MAPE) (%).

Method	DFT	Tersoff1988	Tersoff2007	Tersoff2017	MEAM2007	MEAM2011	SW1985	SW2014

*a*	3.828	3.309	3.820	3.870	4.150	3.837	3.840	3.812
*b*	3.828	3.309	3.820	3.870	4.150	3.837	3.840	3.812
−*E*_c_	4.577	3.936	3.936	3.755	3.404	3.851	3.252	2.572
*d*	2.249	2.315	2.312	2.315	2.534	2.297	2.351	2.243
*h*	0.421	1.304	0.690	0.327	0.820	0.608	0.784	0.427
*C*_11_	69.2	0.006	59.6	50.1	38.4	47.1	36.4	30.8
*C*_22_	69.2	0.006	59.6	50.1	38.4	47.1	36.4	30.8
*C*_12_	22.1	0.002	4.1	5.5	4.4	10.6	6.1	5.4
*C*_33_	23.6	0.002	27.7	22.3	17.0	18.3	15.1	12.7
*K*_I_	91.3	0.008	63.7	55.7	42.7	57.7	42.6	36.2
*K*_II_	47.1	0.004	55.5	44.6	34.0	36.5	30.3	25.5
*K*_III_	47.1	0.004	55.5	44.6	34.0	36.5	30.3	25.5
MAPE_LBS_		79.467	22.781	19.235	37.991	23.569	37.306	35.911

Method		EDIP	ReaxFF	COMB	ML-IAP SNAP	ML-IAP qSNAP	ML-IAP SO(3)	ML-IAP ACE

*a*		4.018	3.843	3.990	3.916	3.808	3.743	3.702
*b*		4.018	3.843	3.990	3.916	3.808	3.743	3.702
−*E*_c_		4.010	3.454	3.911	4.648	4.624	4.606	0.926
*d*		2.321	2.300	2.306	2.390	2.366	2.347	2.242
*h*		0.0^a^	0.610	0.0^a^	0.774	0.859	0.913	0.675
*C*_11_		87.4	49.0	79.7	24.8	28.8	31.7	55.0
*C*_22_		87.4	49.0	79.7	24.8	28.8	31.7	55.0
*C*_12_		9.2	14.1	23.9	5.6	6.3	9.0	2.7
*C*_33_		39.1	17.5	27.9	9.6	11.2	11.4	26.1
*K*_I_		96.6	63.1	103.6	30.4	35.1	40.6	57.7
*K*_II_		78.2	34.9	55.8	19.2	22.4	22.7	52.3
*K*_III_		78.2	34.9	55.8	19.2	22.4	22.7	52.3
MAPE_LBS_		36.630	22.977	19.433	45.306	43.176	42.060	28.792

^a^Input LBS converges to FS.

**Table 4:** Structural and mechanical properties of trigonal dumbbell silicene (TDS) from molecular calculations: lattice parameters *a* and *b* (Å), average cohesive energy *E*_c_ (eV/atom), average bond length *d* (Å), average height *h* (Å), 2D elastic constants *C*_ij_ (N/m), 2D Kelvin moduli *K*_i_ (N/m), and mean absolute percentage error (MAPE) (%).

Method	DFT	Tersoff1988	Tersoff2007	Tersoff2017	MEAM2007	MEAM2011	SW1985	SW2014

*a*	6.434	6.480	6.471	6.475	7.140	6.511	6.600	6.291
*b*	6.434	6.480	6.471	6.475	7.140	6.511	6.600	6.291
−*E*_c_	4.679	4.248	3.865	3.890	3.427	3.865	3.322	2.591
*d*	2.331	2.362	2.371	2.362	2.590	2.376	2.431	2.315
*h*	2.734	2.870	3.110	3.056	3.228	2.856	3.260	3.100
*C*_11_	100.5	79.4	67.9	51.4	69.9	80.5	78.7	68.4
*C*_22_	100.5	79.4	67.9	51.4	69.9	80.5	78.7	68.4
*C*_12_	52.3	63.2	39.4	39.0	34.9	31.0	39.6	35.1
*C*_33_	24.1	8.1	14.3	6.2	17.5	24.8	19.6	16.7
*K*_I_	152.8	142.7	107.3	90.5	104.8	111.5	118.2	103.5
*K*_II_	48.3	16.2	28.5	12.4	35.0	49.5	39.1	33.3
*K*_III_	48.3	16.2	28.5	12.4	35.0	49.5	39.1	33.3
MAPE_TDS_		23.790	22.993	34.815	23.821	11.809	17.077	23.723

Method		EDIP	ReaxFF	COMB	ML-IAP SNAP	ML-IAP qSNAP	ML-IAP SO(3)	ML-IAP ACE

*a*		6.759	6.380	6.574	6.777	6.797	6.518	6.448
*b*		6.759	6.380	6.574	6.777	6.797	6.518	6.448
−*E*_c_		4.075	3.551	3.723	4.726	4.693	4.607	0.4606
*d*		2.416	2.344	2.387	2.398	2.404	2.505	2.429
*h*		2.628	3.115	2.994	2.518	2.540	3.058	2.649
*C*_11_		60.3	94.6	70.8	44.9	36.7	35.3	90.5
*C*_22_		60.3	94.6	70.8	44.9	36.7	35.3	90.5
*C*_12_		30.2	39.7	25.6	16.1	16.7	10.2	33.0
*C*_33_		15.1	27.4	22.6	14.4	10.0	12.5	28.8
*K*_I_		90.5	134.3	96.4	61.1	53.4	45.5	123.5
*K*_II_		30.1	54.9	45.2	28.8	20.1	25.0	57.6
*K*_III_		30.1	54.9	45.2	28.8	20.1	25.0	57.6
MAPE_TDS_		25.537	10.755	16.881	31.929	38.100	37.357	19.317

**Table 5:** Structural and mechanical properties of honeycomb dumbbell silicene (HDS) from molecular calculations: lattice parameters *a* and *b* (Å), average cohesive energy *E*_c_ (eV/atom), average bond length *d* (Å), average height *h* (Å), 2D elastic constants *C*_ij_ (N/m), 2D Kelvin moduli *K*_i_ (N/m), and mean absolute percentage error (MAPE) (%).

Method	DFT	Tersoff1988	Tersoff2007	Tersoff2017	MEAM2007	MEAM2011	SW1985	SW2014

*a*	6.297	6.074^a^	6.133	6.114	5.608^a^	6.364^a^	6.272	6.063
*b*	6.297	5.864^a^	6.133	6.114	6.127^a^	6.279^a^	6.272	6.063
−*E*_c_	4.679	4.334	3.738	3.816	3.663	4.008	3.243	2.467
*d*	2.399	2.398	2.425	2.408	2.641	2.405	2.495	2.394
*h*	2.635	2.596	3.050	3.006	3.500	2.990	3.189	3.010
*C*_11_	141.6	89.3	59.4	64.0	56.2	78.2	57.4	
*C*_22_	141.6	46.3	59.4	64.0	57.1	82.4	57.4	
*C*_12_	96.4	20.4	24.6	31.3	17.3	7.1	25.3	14.9
*C*_33_	22.6	24.8	17.4	16.3	16.7	26.2	16.0	10.4
*K*_I_	238.0	102.1	84.0	95.3	76.6	87.8	82.7	50.6
*K*_II_	45.2	57.7	34.8	32.7	44.5	72.9	32.0	20.7
*K*_III_	45.2	25.5	34.8	32.7	−1.2^b^	52.3	32.0	20.7
MAPE_HDS_		28.361	30.540	29.931	39.880	30.392	33.503	45.400

Method		EDIP	ReaxFF	COMB	ML-IAP SNAP	ML-IAP qSNAP	ML-IAP SO(3)	ML-IAP ACE

*a*		6.349^a^	6.048	6.344	6.644	6.510^a^	5.030^a^	6.250
*b*		6.389^a^	6.048	6.344	6.644	6.344^a^	5.818^a^	6.250
−*E*_c_		4.105	3.366	3.481	4.766	4.699	4.873	0.391
*d*		2.489	2.396	2.511	2.436	2.457	2.612	2.371
*h*		2.365	2.952	2.989	2.404	2.438	2.162	2.559
*C*_11_		78.0	75.4	14.3	41.9	30.8	76.5	69.6
*C*_22_		45.5	75.4	14.3	41.9	16.0	73.0	69.6
*C*_12_		46.3	31.4	−37.4	16.2	13.8	−10.2	10.6
*C*_33_		9.6	22.0	25.8	12.9	11.7	2.7	29.5
*K*_I_		119.7	106.8	51.7	58.1	41.2	84.9	80.1
*K*_II_		13.6	43.9	−23.1^b^	25.7	24.3	64.7	59.0
*K*_III_		9.2	43.9	51.7	25.7	23.4	5.5	59.0
MAPE_HDS_		37.500	22.740	51.780	37.684	41.062	45.571	37.134

^a^Potential does not reproduce the correct symmetry of the structure (a ≠ b), ^b^negative Kelvin moduli *K*_i_ indicating a lack of mechanical stability.

**Table 6:** Structural and mechanical properties of large honeycomb dumbbell silicene (LHDS) from molecular calculations: lattice parameters *a* and *b* (Å), average cohesive energy *E*_c_ (eV/atom), average bond length *d* (Å), average height *h* (Å), 2D elastic constants *C*_ij_ (N/m), 2D Kelvin moduli *K*_i_ (N/m), mean absolute percentage error (MAPE) (%), and relative performance measured as normalized timesteps/second in molecular dynamics (MD) simulation.

Method	DFT	Tersoff1988	Tersoff2007	Tersoff2017	MEAM2007	MEAM2011	SW1985	SW2014

*a*	7.334	7.000^a^	7.249	7.236	7.900^a^	7.363	7.403	7.062
*b*	7.334	6.978^a^	7.249	7.236	7.560^a^	7.363	7.403	7.062
−*E*_c_	4.769	4.468	3.897	4.004	3.505	3.911	3.399	2.602
*d*	2.357	2.381	2.387	2.369	2.627	2.407	2.456	2.345
*h*	2.683	2.692	3.109	3.050	3.050	2.857	3.250	3.100
*C*_11_	104.5	2.3	78.5	68.6	45.6	88.0	84.5	73.0
*C*_22_	104.5	7.3	78.5	68.6	56.2	88.0	84.5	73.0
*C*_12_	52.7	−13.1	42.3	25.5	22.0	29.9	46.1	38.9
*C*_33_	25.9	9.0	18.1	21.6	14.0	29.0	19.2	17.1
*K*_I_	157.2	18.5	120.8	94.1	73.6	117.9	130.6	111.9
*K*_II_	51.8	17.8	36.2	43.1	28.8	58.1	38.4	34.1
*K*_III_	51.8	−8.5^b^	36.2	43.1	27.4	58.1	38.4	34.1
MAPE_LHDS_		55.693	18.373	20.287	34.499	13.636	16.741	23.855

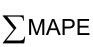		223.826	129.306	150.256	164.364	82.553	130.721	155.483

timesteps/s		387.2	382.7	355.2	505.7	416.9	904.9	1753.8

Method		EDIP	ReaxFF	COMB	ML-IAP SNAP	ML-IAP qSNAP	ML-IAP SO(3)	ML-IAP ACE

*a*		7.705	7.167	7.422	7.648	7.741	7.427	7.393
*b*		7.705	7.167	7.422	7.648	7.741	7.427	7.393
−*E*_c_		4.113	3.623	3.646	4.804	4.794	4.698	0.370
*d*		2.438	2.370	2.454	2.436	2.403	2.385	2.515
*h*		2.637	3.112	2.994	2.538	2.562	2.700	2.712
*C*_11_		53.7	99.0	53.1	44.6	43.8	25.9	59.8
*C*_22_		53.7	99.0	53.1	44.6	43.8	25.9	59.8
*C*_12_		36.0	40.4	31.7	16.1	19.1	0.4	15.2
*C*_33_		8.8	29.3	10.7	14.2	12.3	12.7	22.3
*K*_I_		89.7	139.4	84.8	60.6	62.8	26.4	75.0
*K*_II_		17.6	58.6	21.5	28.5	24.7	25.5	44.7
*K*_III_		17.6	58.6	21.5	28.5	24.7	25.5	44.7
MAPE_LHDS_		33.228	10.809	33.466	33.219	34.603	40.928	29.310

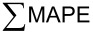		165.722	78.185	137.365	181.422	205.224	201.861	124.310

timesteps/s		2032.1	23.6	26.4	7.9	4.7	4.2	1.0

^a^Does not reproduce the correct symmetry of the structure (a ≠ b), ^b^negative Kelvin moduli *K*_i_ indicating a lack of mechanical stability.

## Conclusion

A systematic quantitative comparative study of various silicon interatomic potentials for reproducing the properties of five silicene (2D silicon) polymorphs was shown. In order to compare the fourteen potentials listed in section “Interatomic potentials”, the structural and mechanical properties of flat (FS), low-buckled (LBS), trigonal dumbbell (TDS), honeycomb dumbbell (HDS), and large honeycomb dumbbell (LHDS) silicene (Figure 1) obtained from density functional theory (DFT) and molecular statics (MS) computations were used. Computational cost and performance of the analyzed potentials were compared.

Considering the performance and the cost of calculations, the classical potentials of Tersoff, SW, and MEAM types seem to be the best choice here. Although data for silicene polytypes were not used in the optimization of these potentials, they were able to reproduce their properties well. This is a consequence of the fact that they are based on physics, have a natural extrapolation ability, and do not just interpolate data.

I hope that the findings presented here will help other researchers in selecting the suitable potentials for their purposes and will be a hint to parameterize new potentials for silicene.

## Supporting Information

Crystallographic Information Files (CIF) for polymorphs of silicene (created by qAgate (Opensource software to post-process ABINIT) and then converted to P1 setting by Bilbao Crystallographic Server [48–49]).

File 1 Flat silicene (FS).

File 2 Low-buckled silicene (LBS).

File 3 Trigonal dumbbell silicene (TDS).

File 4 Honeycomb dumbbell silicene (HDS).

File 5 Large honeycomb dumbbell silicene (LHDS).
